# Episomal vectors based on S/MAR and the β-globin Replicator, encoding a synthetic transcriptional activator, mediate efficient γ-globin activation in haematopoietic cells

**DOI:** 10.1038/s41598-019-56056-z

**Published:** 2019-12-24

**Authors:** Eleana F. Stavrou, Emannuouil Simantirakis, Meletios Verras, Carlos Barbas, George Vassilopoulos, Kenneth R. Peterson, Aglaia Athanassiadou

**Affiliations:** 1 Department of General Biology, School of Medicine, University of Patras, Patras, Greece; 2 Hematology Clinic, Medical School, University of Thessaly and Gene and Cell Therapy Laboratory, BRFAA, Athens, Greece; 3 Skaggs Institute for Chemical Biology, Department of Molecular Biology, Scripps Research Institute, La Jolla, California USA; 4 Department of Biochemistry and Molecular Biology, University of Kansas Medical Center, Kansas City, Kansas USA

**Keywords:** Biotechnology, Gene delivery, Genetic vectors

## Abstract

We report the development of episomal vectors for the specific γ-globin transcription activation in its native position by activator Zif-VP64, based on the Scaffold/Matrix Attachment Region (S/MAR) for episomal retention and the *β-globin Replicator*, the DNA replication-Initiation Region from the β-globin locus. Vector Zif-VP64-Ep1 containing transcription cassettes CMV- Zif-VP64 and CMV-eGFP-S/MAR transfected a)K562 cells; b)murine β-YAC bone marrow cells (BMC); c)human haematopoietic progenitor CD34^+^ cells, with transfection efficiencies of 46.3 ± 5.2%, 23.0 ± 2.1% and 24.2 ± 2.4% respectively. K562 transfections generated stable cell lines running for 28 weeks with and without selection, with increased levels of γ-globin mRNA by 3.3 ± 0.13, of γ-globin protein by 6.75 ± 3.25 and HbF protein by 2 ± 0.2 fold, while the vector remained episomal and non integrated. In murine β-YAC BMCs the vector mediated the activation of the silent human γ-globin gene and in CD34^+^ cells, increased γ-globin mRNA, albeit only transiently. A second vector Zif-VP64-Ep2, with both transcription cassettes carrying promoter SFFV instead of CMV and the addition of *β-globin Replicator*, transferred into CD34^+^ cells, produced CD34^+^ eGFP^+^ cells, that generated colonies in colony forming cell cultures. Importantly, these were 100% fluorescent, with 2.11 ± 0.13 fold increased γ-globin mRNA, compared to non-transfected cells. We consider these episomal vectors valid, safer alternatives to viral vectors.

## Introduction

Episomes, in eukaryotes, are extrachromosomal, closed circular DNA molecules of a plasmid or a viral genome origin, that are replicated autonomously in the host cell and therefore, they bear significant vector potential for the transfer of nucleic acids into cells. Such is the case of the Replicating Episomal Vectors, that have been engineered^1^ and used for the study of gene expression and in gene therapy applications^2^, however, the efficacy of early episomal gene transfer was limited by transient gene expression, mainly due to inefficient mitotic segregation and plasmid loss. A milestone in the development of stably maintained episomal vectors was the production of the vector pEPI-1^3^, a 6.69 kb plasmid, which does not code for any viral protein and it is capable for long-term nuclear retention and transgene expression in the recipient cell. Vector pEPI-1 is based on the SV40 origin of replication, with the SV40-encoded gene for large T-antigen being replaced with the chromosomal element Scaffold/Matrix Attachment Region (S/MAR). The chromosomal elements termed S/MARs, are AT rich regions of the genome, that play a role in chromatin boundary formation^4,5^, they bind to *scaffold attachment factor A (*SAF-A) protein^6^, and they mediate tethering of pEPI-1 plasmid to the nuclear matrix, thus promoting their nuclear retention. The S/MAR element that is present in plasmid pEPI-1 derives from the 5′ end of the human β-interferon gene and must be transcribed to exert its nuclear retention function. Vector pEPI-1 undergoes episomal replication once per cell cycle, at low copy number, synchronously with the cellular DNA and with very high degree of mitotic stability^7^, due to the anchoring function of the transcribed S/MAR element. Plasmid pEPI-1 and derivatives function as efficient episomal vectors *in vitro*, in cell lines^8^ and primary cell cultures^9^ as well as *in vivo*, in the mouse liver^10^ and in genetically modified organisms^11^ and show ability to support safe and reproducible genetic modification of cells in gene therapy applications^12–14^. Thus, the S/MAR-based pEPito plasmid designed for episomal persistence has been demonstrated to be efficient in *in vitro* and *in vivo* studies^15^, while recently, S/MAR-stabilized plasmids encoding the Cystic Fibrosis Transmembrane Conductance Regulator (CFTR) gene, were added to the tools used in the long standing quest for Cystic Fibrosis gene correction^16^. The ‘S/MAR technology’ emerging from episomal vectors has also been combined with viral delivery systems, to produce vectors that are superior to either system alone. Such are, for example, the hybrid-vectors based on high-capacity adenoviral vectors for efficient delivery of autonomous replicons^17^ and the long-term stabilization, by the use of S/MAR, of the non-integrating lentiviral vector (NILV)^18^.

Episomal vectors, may prove to be efficient for the treatment of difficult inherited diseases, such as β-thalassaemia and sickle cell disease (SCD), both caused by mutations in the β-globin gene, providing a new platform for gene therapy of these diseases. Currently, the most advanced gene therapy approach for these diseases involves the use of complex lentiviral vectors, that harbour a functional β-globin gene, to modify haematopoietic stem cells of the patient^19^. After initial failures, the use of self-inactivating (SIN) lentiviral vectors that are significantly less genotoxic^20,21^ led to the first successful gene therapy for β-thalassaemia^22^. Since then, a number of ongoing clinical trials continue to evaluate the efficacy of lentiviral vectors that carry β-like globin transgenes for modification of autologous β-thalassaemic and SCD HSCs^23,24^.

A second strategy for β-thalassaemia and SCD gene therapy, and of relevance to this work, aims at increasing fetal haemoglobin (HbF) which consists of two α-globin and two γ-globin chains, by activating the γ-globin gene(s), a member of the β-like gene cluster. Most β-thalassaemia mutations or SCD mutations in the β-globin gene lead to a decrease, absence, or functional alteration of the adult haemoglobin (HbA), which consists of two α-globin chains and two β-globin chains.The activation of a γ-globin gene, normally expressed in fetal life, during the adult stage of the patient, compensates for the loss of β-globin gene expression or function, and is a well-validated therapeutic option, based on the amelioration of the clinical phenotype of β-thalassaemia and SCD patients through the presence of high HbF, particularly within the Hereditary Persistence of Fetal Haemoglobin (HPFH) syndrome^25^. These data prompted research originally on the pharmacological induction of γ-globin gene transcription in the HSC of patients^26,27^ and later on the formulation of molecular therapy aiming at reversing the action of endogenous repressors of γ-globin gene transcription^28^.

With the advent of ‘genome editing’ technology, research focused (a) on correcting the mutations that cause β-haemoglobinopathies in their native position within the β-globin locus^29^, (b) on the activation of γ–globin genes by silencing of transcription factors that repress its transcription^30,31^, or (c) on the introduction of the HPFH genotype into HSCs^32^. The potential of these approaches for clinical application is currently under intense investigation^33,34^. Other avenues in this treatment category, for example, using promotorless genes without nucleases, also appear promising^35^.

While integrating lentiviral vectors have become the preferred platform for gene therapy in haematopoietic disorders^36^, the residual oncogenic potential by the integration of these vectors raises concerns, as even a single insertion event is sufficient to initiate a cascade of events resulting in leukemic transformation *in vivo*^37,38^. Indeed, recently a first clear link was presented, between lentiviral insertion-induced clonal expansion and a clinically abnormal transformed phenotype in a primate, following transduction of human HSPC with LV with a strong promoter^39^. The use, previously, of moderate promoters and the presence of a synthetic chromatin insulator cassette did not alleviate vector-mediated genotoxicity^40^, but proper design of powerful insulators^30^, as well as the possibility of monitoring vector integration sites^41,42^ may improve their performance.

However, insertional mutagenesis is not the only problem with the lentiviral vectors in use, as integration competent LV and non-integrating LV episomes as well as AAV DNA can trigger the tumor suppressor p53 gene signaling in human haematopoietic stem and progenitor cells^43^.

Within the concept of developing alternative, episomal, S/MAR-based vectors for the haemoglobinopathies, we made use of another chromosomal element, a *bona fide* mammalian origin of replication, namely the *β-globin Replicator*, which is the replication-Initiation Region (IR) from the human β-globin locus, containing the 1.3 kb that represents the consensus ‘IR’ region. This region is considered to be a Replicator because of its capacity to initiate DNA replication in ectopic sites^44^. This IR element was used twice in the past along with the S/MAR element, to produce episomal vectors with positive results^9,45^. In this work, we combine episomal gene transfer with trans-activation of a γ-globin gene. We report the construction and characterization of episomal vectors, based on S/MAR element, with or without the IR element, that carry an artificial transcription activator, gg1-VP64, for specifically trans-activating the human ^A^γ-globin gene^46^ in human and murine haematopoietic cells. In the past, viral delivery of this artificial activator of the ^A^γ-globin gene, gg1-VP64-HA, into a variety of target cells, of human^46^ or murine origin^47,48^ as well as the CD34^+^ progenitor cells from normal human donors and β-thalassaemia patients^49,50^ resulted in selective and direct interaction with the promoter of the endogenous ^A^γ-globin gene and upregulation of its transcription. Thus, this system may contribute towards an effective gene therapy for treatment of β-thalassaemia and sickle cell disease.

## Results

The specific aim of this work was to investigate the potential of newly designed episomal vectors, based on the S/MAR and the IR elements, to activate the transcription of the γ-globin gene in its native position, by the synthetic activator VP64, in cells of hematopoietic origin. To this end, the vector was tested: **(i)** in the erythroleukemia cell line K562, in order to set transfection parameters and test the capacity for episomal long term retention, expression maintenance and vector copy number dependent expression, **(ii)** in cells from transgenic mice carrying the human β-YAC, in which the γ-globin gene has been silenced, in order to test the capacity of the vector to activate this gene from within heterochromatin region and **(iii)** in human CD34^+^cells in order to determine the efficacy of transfection and the functional potential of the vector with the very cells that are the target for therapeutic gene transfer in these diseases.

### The design of the Vectors

**Figure 1:**
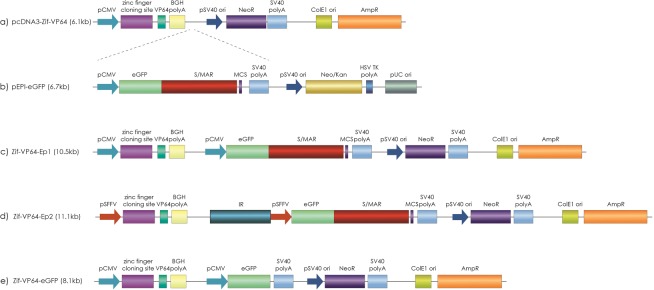
Plasmid constructs. (**a**) Plasmid pcDNA3-Zif-VP64 (6.1 kb), (**b**) Control plasmid pEPI-eGFP (6.7 kb), (**c**) Newly developed plasmid Zif-VP64-Ep1(10.5 kb) that contains two individual expression cassettes pCMV-eGFP-S/MAR and pCMV-Zif-VP64, (**d**) control plasmid Zif-VP64-eGFP (8.1 kb) that lacks S/MAR sequence, (**e**) newly developed plasmid Zif-VP64-Ep2 (11.1 kb) that contains two individual expression cassettes IR-pSFFV-eGFP-S/MAR and pSFFV-Zif-VP64.

Plasmids pcDNA3-Zif-VP64 (Fig. 1a)^46^ and pEPI-eGFP (Fig. 1b)^51^ supplied the basic transcription units, namely one for the γ-globin activator Zif-VP64 and one for the reporter gene eGFP respectively, that are contained in the newly constructed vectors. Specifically, the episomal vectors used for γ-globin activation in this work were of two kinds.The first vector constructed, Zif-VP64-Ep1 (Fig. 1c), carries the two transcription cassettes, one for the specific γ-globin activator CMV-Zif-VP64 and one for the reporter gene CMV-eGFP-S/MAR, both driven by promoter CMV. The cassette CMV-Zif-VP64 carries the domain VP64, a tetrameric repeat of the minimal activation domain of the herpes simplex protein VP16, while the Zinc finger (Zif) part of this activator is a DNA binding domain, that binds specifically the promoter on the ^A^γ-globin gene, at a 17 nucleotides long area, designated gg1, around the −117 position^46^, which harbours a naturally occurring HPFH site^52,53^. The cassette CMV-eGFP-S/MAR carries the reporter eGFP gene followed by the S/MAR element, deriving from the 5′ position of the human beta-interferon gene, so that transcription runs through the S/MAR, a necessary condition for nuclear retention element of the plasmid by the S/MAR element.

The second vector constructed, Zif-VP64-Ep2 (Fig. 1d), carries the same two transcription cassettes of the Zif-VP64-Ep1 vector, but promoter CMV was replaced by promoter SFFV in both cases, to ensure transcription in the CD34^+^ cells. Additionally, this vector carries the new chromosomal element, the *β-globin Replicator*, or ‘IR’, which is the replication-Initiation Region (IR), deriving from the human β-globin cluster, and it is added in order to enhance the plasmid’s replication capacity^9,45^.

Vector Zif-VP64-eGFP (Fig. 1e) was constructed to be devoid of both the S/MAR and IR elements, was used as control along with vector Zif-VP64-Ep1 in the K562 transfections.

### Vector Zif-VP64-Ep1 stably transfects K562 cells

Plasmid Zif-VP64-Ep1 was used to transfect cells from a growing cell culture of the human myelogenous leukaemia cell line K562, in order to generate stably transfected pools of cells. K562 cells constitutively express the embryonic ε- and fetal γ-globin, but not the adult β-globin gene^54^. The aim, therefore, is to test for an increase of the fetal γ-globin, in transfected, long-term cultures.

**Figure 2:**
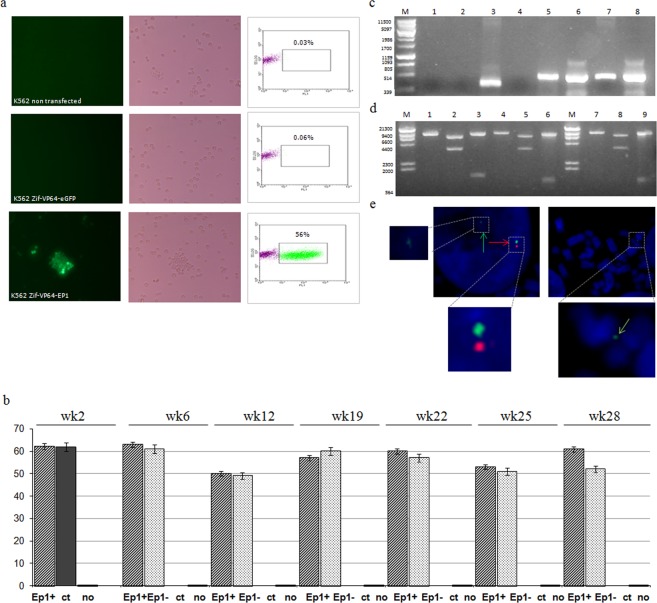
Documentation of eGFP expression and episomal status of vector Zif-VP64-Ep1 in transfected K562 cells. All experiments were carried out in triplicate. (**a**) K562 cells non transfected 19-weeks post transfection (upper panel photos), K562 cells transfected with Zif-VP64-eGFP control vector, 6-weeks post transfection (middle panel photos) and K562 cells transfected with Zif-VP64-Ep1 plasmid, 19-weeks post transfection (−G418) (down panel photos). Results are shown from fluorescent microscopy (left column), phase contrast (middle column) and flow cytometry, giving the estimated percentage of fluorescent, transfected cells (right column). y-axis records counts of cells and x-axis records FL1-eGFP fluorescence. (**b**) Histogram demonstrating expression eGFP based on flow cytometer measurements. (Ep1) K562 cells transfected experimental vector Zif-VP64-Ep1, K562 cells cultured (+) with or (−) without selection pressure (G418) at various times after transfection as indicated (week 2, 6, 19, 22, 25, 28); (no) indicates non transfected K562 cells and (ct) K562 cells transfected with the Zif-VP64-eGFP control vector. (**c**) PCR on DNA extracts from K562 cells (6 weeks post transfection) (M) Phage Lambda DNA- *Pst I*; All four 1, 2, 3 and 4 runs refer to PCR reactions with eGFP primers (amplified fragment size 350 bp) and all four 5, 6, 7 and 8 runs refer to PCR control reactions with β-globin primers (amplified fragment size 453 bp); (1, 5) K562 cells non transfected; (2, 6) and (4, 8) K562 cells transfected with Zif-VP64-eGFP, from two different PCR reactions; (3, 7) K562 cells transfected with Zif-VP64-Ep1. (**d**) Plasmid rescue experiment with restriction analysis. Zif-VP64-Ep1 DNA vector used as input for transfections (7, 8, 9) and DNA prepared from a colony of *E. coli* transformed with a HIRT extract of Zif-VP64-Ep1 rescued plasmid from transfected K562 cells 19 weeks after transfection (1, 2, 3, 4, 5, 6). Restriction enzymes used were *NotI*, *PciI*, *NheI*. (1, 4, 7) stands for restriction with *NotI*, (2, 5, 8) with both *NotI* and *PciI*, (3, 6, 9) with *NheI* (M) stands for *λHindIII* DNA marker in both cases. (**e**) K562 cells transfeced with vector Zif-VP64-Ep1, after 22 weeks of continuous culture supplemented by G418 were analyzed by fluorescent *in situ* hybridization (FISH). Representative interphase (left) and metaphase (middle) spreads are shown. Green arrows show the Zif-VP64-Ep1 plasmid as episomes in non-integrated status and red arrow show the control endogenous 13q14 locus giving a double -green and red–signal.

Triplicate transfections were carried out with vector Zif-VP64-Ep1 and the control plasmid Zif-VP64-eGFP and transfection efficiency was on average 46.3 ± 5.2% (Fig. 2a) and 48.1 ± 1.3% respectively. Twenty-four hours after transfection, selection with Geneticin (G418) was applied and two weeks later transfected cultures for both Zif-VP64-Ep1 and control vectors were obtained. At this point cultures were split in two parts and one part was kept under continuous selection (G418+) while the other part was kept under no selection (G418−). Non transfected cells were cultured in parallel, with and without selection. Cell cultures growing without selection, were supplemented with G418 and kept for one further week, in order to test for G418 resistance.

Results are shown in Fig. 2 and, firstly, it is established that non transfected cells do not grow in the presence of G418 (Fig. 2a,b). Cells carrying the control plasmid Zif-VP64-eGFP are selected by the application of G418 (see Methods for details), but after the cultures were split, the part that was kept under selection does not generate stably transfected, long term cell culture; instead, it is gradually dying out and effectively it is extinct by week 6. The part of the culture that was growing in the absence of G418, continued to grow, but its fluorescence was lost by week 6. Upon the application of G418, at week 6, these cultures died out as devoid of plasmids (Figure S1 in Supplementary Information). At the same time PCR was carried out on DNA from cells growing in the absence of G418, using primers for eGFP, and no vector plasmid was detected (Fig. 2c), showing that the cells were not fluorescent because they were devoid of vector and not because of transgene silencing.

In contrast, cells carrying plasmid Zif-VP64-Ep1 form fully growing, stably transfected cultures, with and without G418 selection (Fig. 2b).This is documenting that the S/MAR element is necessary and adequate part of the vector, for the formation of stable, long term culture in K562 cells.

The cells that had grown without selection for long time, were found to be still resistant to G418 at week 28 of culture (or (Fig. 2b). These data show that the S/MAR based plasmid Zif-VP64-Ep1 is capable for supporting stable, long-term culture of transfected K562 cells. This is the first step towards the development of an episomal vector for the trans–acting γ-globin activator VP64.

### The episomal status of Vector Zif-VP64-Ep1 in K562 cells

Stably transfected K562 cells with vector Zif-VP64-Ep1 were used to determine the status of this vector as an episome, in the transfected cells. Plasmid rescue assay was performed to detect free vector plasmids, using plasmid DNA prepared by Hirt extract from duplicate cultures for 5, 19 and 28 weeks. The DNA of the Hirt extract was used to transfect competent *E.coli* cells, and colonies (3–5 in each case) appeared, which shows that intact, circular plasmid molecules were contained in the Hirt extract. Results shown for the 19^th^ week of tranfected K562 culture (Fig. 2c), reveal that the DNA from the *E.coli* colonies has the same restriction pattern as does the input DNA of vector Zif-VP64-Ep1, used for transfections of K562 cells, and it is therefore the same plasmid. This shows that the vector Zif-VP64-Ep1 is maintained as a free, circular DNA molecule, capable for autonomous replication with stable mitotic segregation within the respective, transfected K562 cell culture.

Fluorescent *In Situ* Hybridization (FISH) was applied to investigate the possibility of integration of vector Zif-VP64-Ep1 into the endogenous genetic material of the K562 cells. Results show (Figs. 2d and S2 in Supplementary Information) no detection of integration events, that would be detected as a clone of twin signal for the plasmid in the same chromosome, in over 50 metaphase cells that were examined, as only one or two copies per cell were detected in random positions. This leads to the conclusion that data based on the eGFP fluorescent derive from free circular DNA molecules rather than from clones of integrated plasmid copies.

### Estimation of the vector copy number per cell

This is important as it provides a measure of the vector’s potential. For this estimation we applied the Absolute Quantification analysis (see Materials and Methods) and the plasmid copy number was found to be 2.4 ± 0.2 per cell for the cultures growing in G418 + condition at the 16^th^ week of culture. This is within the expected range for the size of the plasmid^9^. Additionally, FISH analysis (Figs. 2e and S2 in Supplementary Information) shows the existence of one or two plasmid copies per cell, in accordance with this result.

### Vector Zif-VP64-Ep1 supports the production of the specific, functional activator

The expression of the transgene for the γ-globin activator VP64 from within vector Zif-VP64-Ep1 was investigated in stably transfected K562 cells.

**Figure 3:**
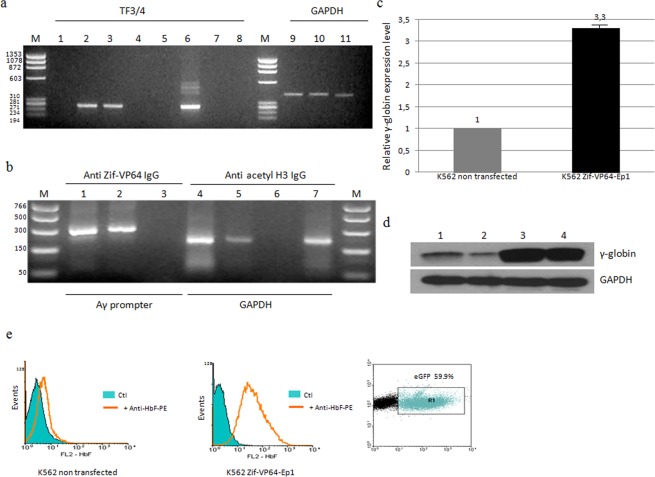
Expression of transgene and induction of γ-globin gene in K562 cells. All experiments were carried out in triplicate. Documentation of Zif-VP64 transgene expression in transfected K562 cells: (**a**) Electrophoresis of RT-PCR reaction products (TF3/TF4) with the TF3/TF4 primers in nested PCR. (1) K562 cells, (2) K562 transfected with the vector Zif-VP64-Ep1 +G418, (3) K562 transfected with the vector Zif-VP64-Ep1 −G418, (4) Zif-VP64-Ep1 +G418 without reverse transcriptase, (5) Zif-VP64-Ep1 −G418 without reverse transcriptase, (6) Zif-VP64-Ep1, (7) and (8) H_2_O. (GAPDH) Electrophoresis of RT-PCR reaction products with the GAPDH-F/R primers (9) K562 cells, (10) K562 transfected with the vector Zif-VP64-Ep1 +G418, (11) K562 transfected with the vector Zif-VP64-Ep1 −G418. (M) FX/HaeIII Digest in both cases. Certification of the specific binding of Zif-VP64 transcription factor to the γ-globin promoter: (**b**) Agarose gel electrophoresis of PCR products from: (Anti-Zif-VP64 IgG) immunoprecipitated chromatin with anti-Zif-VP64 IgG and amplified with Chip_F/R primers for part of the γ-globin promoter, from transfected K562 cells with Zif-VP64-Ep1 vector, 3 weeks after transfection (1) +G418 and (2) –G418, (3) non transfected K562 cells; (Anti acetyl H3 IgG) chromatin immunoprecipitated with anti-acetyl Histine H3 IgG and amplified with GAPDH_F/R primers for the GAPDH gene from transfected K562 cells with Zif-VP64-Ep1 vector, 3 weeks after transfection; (4) +G418 and (5) −G418, (6) PCR without DNA matrix, (7) non transfeced K562 cells and (M) PCR marker NEB. Induction of γ-globin in K562 cells transfected with Zif-VP64-Ep1: (**c**) γ-globin mRNA expression in K562 cells transfected with Zif-VP64-Ep1 plasmid (K562 Zif-VP64-Ep1) within the 4 to 10 weeks period of continuous culture, as estimated by RT-qPCR. Values were normalized against non transfected K562 cells (K562), which was taken as 1; (**d**) γ-globin protein derived from K562 cells transfected with Zif-VP64-Ep1plasmid analyzed with Western blot analysis. (1, 2) K562 non transfected cells, K562 cells transfected with Zif-VP64-Ep1 (3) 5 weeks and (4) 15 weeks period of continuous culture. (**e**) HbF protein derived from K562 cells transfected with Zif-VP64-Ep1 plasmid (K562 Zif-VP64-Ep1) (left column) estimated by intracellular staining with flow cytometry at week 15 period of continuous culture.Values were normalized against non transfected K562 cells (K562) (middle column). eGFP expression in transfected K562 cells Zif-VP64-Ep1 at week 15, as documented with flow cytometry giving the estimated percentage of fluorescent eGFP^+^, transfected cells (right column). y-Axis records counts of cells and x-axis records FL1-eGFP fluorescence.

Results are positive for the presence of this activator, as mRNA for Zif-VP64 was detected in total RNA isolated from transfected cells every 3 weeks from transfection, by one step RT-PCR, and a representative experiment is shown for the 21^st^ week (Fig. 3a). Furthermore, the ability of the Zif-VP64 peptide to bind its target position, namely the ‘gg1’ site in the promoter of the ^A^γ-globin gene, in K562 cells (3weeks post transfection) was tested in two independent transfections by chromatin immunoprecipitation assay (ChIP) (Fig. 3b). This was carried out by the use of specific antibodies for Zif-VP64, followed by a PCR reaction, using specific primers for the binding DNA sequence at the ^A^γ-gene promoter. It is herein documented that the Zif-VP64 peptide, produced by the episomal vector, binds to ^A^γ-globin promoter, thus fulfilling the necessary pre-condition for γ-globin activation.

### Episomal vector Zif-VP64-Ep1 mediates efficient induction of γ-globin in K562 cells

The K562^54^ cells are triplicate for chromosome 11 and are expressing γ-globin. The transfer of plasmid Zif-VP64-Ep1, potentially resulting in γ-globin induction, is expected to bring about an increase in the amount of steady state γ-globin mRNA in these cells. The data obtained clearly show that there is definite increase in the γ-globin expression in transfected versus non transfected cells in all cases tested. The γ-globin mRNA produced, evaluated by RT-qPCR, had 3.3 ± 0.13fold increase (Fig. 3c); the γ-globin peptide, detected by western blot analysis (14^th^ week) (Figs. 3d and S3 in Supplementary Information) had a 6.75 ± 3.25 fold increase (quantitative results of western blot films by Image J application) and the HbF protein estimated by intracellular staining (Fig. 3e) at week 15, had 2 ± 0.2 fold increase.

It is therefore demonstrated that the episomal vector Zif-VP64-Ep1 has the capacity for long-term support of the production of a functional inducer of the γ-globin gene in K562 cells.

### The episomal vector Zif-VP64-Ep1 activates the γ-globin gene within the β-YAC transgenic murine cells

In the next round of experiments, we used murine primary hematopoietic progenitor cells containing the entire human β-globin gene locus (248 kb) as a yeast artificial chromosome^47^.This is a multipotent cell line that upon addition of the appropriate growth factors, can generate monocytes, neutrophils and erythroid cells while cultures can be maintained for a year or more. The murine β-YAC transgenic cells present the adult human profile of β-globin locus gene expression, in that they express the β-globin gene but not γ-globin gene. The lack of the γ-globin mRNA in these cells was also documented in this study, by RT-qPCR (Figure S4 in Supplementary Information). They are, therefore, a good model for testing the reactivation of a silent γ-globin gene. To this purpose, transfections in murine β-YAC transgenic cells are here studied only for maximum 7 days and, therefore, cannot be used to evaluate the role of the S/MAR in these vectors-cells condition.

**Figure 4:**
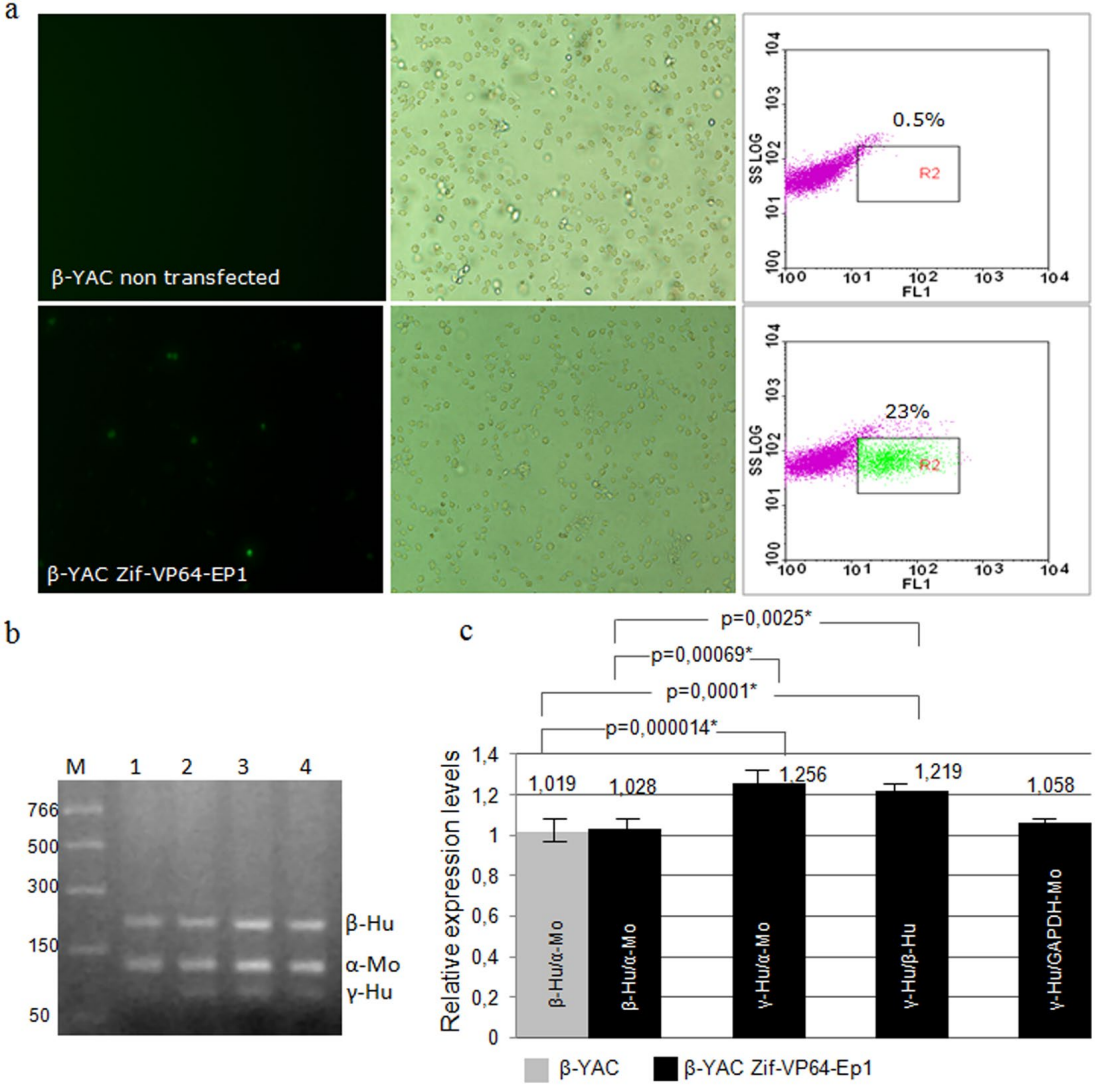
Documentation of eGFP expression and activation of human γ-globin in β-YAC cells transfected with Zif-VP64-Ep1. (**a**) β-YAC cells non transfected (upper panel photos) and β-YAC cells transfected with Zif-VP64-Ep1 plasmid 4 days post transfection (down panel photos). Results are shown from phase contrast (right column), fluorescent microscopy (left column) and from flow cytometry, giving the estimated percentage of fluorescent, transfected cells (right column); y-axis records counts of cells and x-axis records FL1-eGFP fluorescence; (**b**) multiplex RT-PCR for human γ-globin (γ-Hu), humanβ-globin (β-Hu) and mouse α-globin (α-Mo) mRN; (1) non transfected β-YAC cells, (2, 3, 4) β-YAC cells transfected with Zif-VP64-Ep1 after 2, 4 and 7 days of culture and (M) PCR marker NEB; (**c**) RT-qPCR for human γ-globin (γ-Hu), humanβ-globin (β-Hu) and mouse α-globin (α-Mo) mRNA expression in β-YAC cells transfected with Zif-VP64-Ep1, 7 days of culture post transfection (β-YAC Zif-VP64-Ep1) normalized over non transfected β-YAC (β-YAC). Results of statistical significance are shown, (all <0.05 value).

Transfections in 4 replications of the murine β-YAC transgenic cells were performed with plasmid Zif-VP64-Ep1 and transfection efficiency was 23.0 ± 2.1% (Fig. 4a). Multiplex RT-PCR was applied for a first, qualitative documentation of (a) the γ-globin reactivation in the transfected cells along with the human β- and mouse α-globin mRNAs, and (b) the absence of γ-globin mRNA in the non transfected cells. Reactivation of the γ-globin mRNA was detected in these cells after 2, 4 and 7 days (Fig. 4b).

For the estimation of γ-globin expression in the transfected, murine B-YAC transgenic cells, RT-qPCR was applied and the relative amount of the γ-globin mRNA against that of human β-globin and mouse α-globin one was determined. Results show that the level of the γ-globin mRNA produced in these cells exceeded those of the human β-globin mRNA or the mouse α-globin mRNA by about 21.9 ± 8.4% and 25.6 ± 3.9% respectively, and these values were statistically significant (Fig. 4c). The mRNAs of the human β-globin mRNA and the mouse α-globin mRNA were produced in rather equal amounts (Fig. 4c). These results show that the episomal vector Zif-VP64-Ep1 can support the reactivation of a silent γ-globin gene, to a degree that may signify a therapeutic level of the γ-globin peptide produced.

### The episomal vectors induce the γ-globin gene in human CD34^+^ cells

Human heamatopoietic progenitor CD34^+^ cells are producing γ-globin mRNA. They were isolated here from total mononuclear cells, from cord blood and were used in transfections by nucleofection. Transfections in CD34 ^+ ^cells with each vector were carried out in 4 replications. Control vector Zif-VP64-eGFP is capable of transfecting CD34 + cells with efficiency 21.8 ± 4.0%, measured at 48 hours post transfection, but, contrary to its transfections in K562 cells, cells were non fluorescent 24 hours later, total 72 hours post transfection (Table 1). Therefore, this vector could not be used as a control for colony forming assays (CFC) with the CD34^+ ^cells.

**Table 1:** Colony Forming Cell assay giving rise to BFU-E and CFU-GM differentiated colonies from CD34^+^ hematopoietic progenitor cells, transfected with vectors Zif-VP64-eGFP, Zif-VP64-Ep1 and Zif-VP64-Ep2.

	% eGFP^+^ 48h*#	expression eGFP, 72h*	CFCs from FACS-sorted CD34^+^/eGFP^+^ cells 14days post sorting											
			BFU-E				CFU-GM				Total			
			n	µ	µ	s.d (%)	n	µ	µ	s.d (%)	n	µ	µ	s.d (%)
Zif-VP64-eGFP	21.8 ± 4.0%	eGFP^-^		__				__				__		
Zif-VP64-Ep1	24.2 ± 2.4%	eGFP^+^	0/50	0/30	0	0	0/8	0/4.5	0	0	0/58	0/34.5	0	0
			0/35				0/4				0/39			
			0/25				0/2				0/27			
			0/10				0/4				0/14			
Zif-VP64-Ep2	23.3 ± 1.7%	eGFP^+^	11-Nov	18.25/18.25	100	0	03-Mar	03-Mar	100	0	14/14	21.5/21.5	100	0
			13/13				05-May				18/18			
			20/20				03-Mar				23/23			
			30/30				01-Jan				31/31			

*Post transfection; ^#^transfection efficiency; eGFP positive colonies in CFCs derived from.

FACS-sorted CD34^+^/eGFP^+^ cells; transfected with vectors Zif-VP64-Ep1 and Zif-VP64-Ep2;

n depicts the fraction of eGFP^+^ colonies over total number of colonies per dish from 4 transfection.

experiments; μ depicts the average fraction of these experiments.

BFU-E: Burst-Forming-Unit-Erythroid, primitive erythroid progenitor cells,

CFU-GM: Colony Forming Unit-Granulocyte-Monocyte, precursor of monoblasts and myelonblasts.

**Figure 5:**
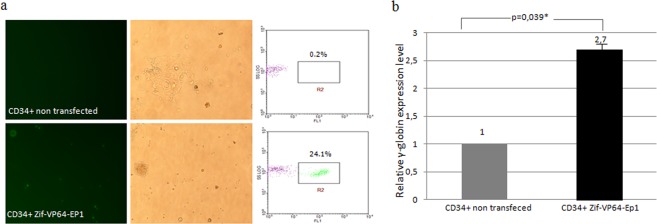
Documentation of eGFP expression and induction of γ-globin in CD34+ cells transfected with Zif-VP64-Ep1. Experiments were carried out in 4 duplicates. (**a**) eGFP expression depicted at 48 hours post transfection and prior to FACS sorting; from fluorescent microscopy (left column) and phase contrast (middle column) and from flow cytometry, giving the estimated percentage of fluorescent CD34^+^/eGFP^+^ transfected cells (right column). y-Axis records counts of cells and x-axis records FL1-eGFP fluorescence; (**b**) γ-globin mRNA expression in CD34^+^ transfected cells with Zif-VP64-Ep1 plasmid (CD34^+^ Zif-VP64-Ep1) 72 hours post transfection −24 hours after FACS sorting- as estimated by RT-qPCR. Values were normalized against non transfected CD34^+^ cells, which was plotted as 1.

**Figure 6:**
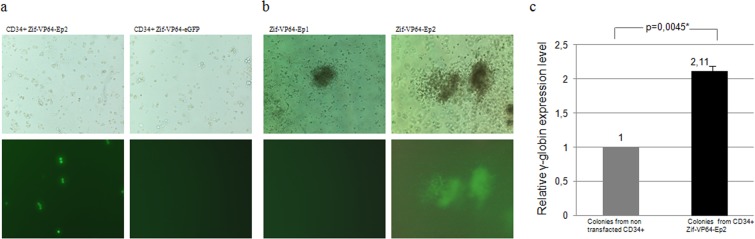
Results from transfection into CD34+ cells of vectors Zif_VP64-eGFP, Zif-VP64-Ep1 and Zif-VP64-Ep2. Transfected CD34+ cells were sorted by FACS 48 hours post transfection. (**a**) eGFP expression as documented in CD34^+^ cells carrying Zif-VP64-Ep2 (right) and Zif VP64-eGFP vectors (left) 72 hours post transfection. Results are shown from phase contrast (upper column) and fluorescent microscopy (lower column). (**b**) Documentation of eGFP expression in colonies derived from CFC assays in semisolid cultures (14 days) of FACS sorted CD34^+^/eGFP^+^ cells from CD34^+^ cells, 72 hours post transfection with Zif-VP64-Ep1 control vector (left) or with Zif-VP64-Ep2 vector (right).Upper row: Colonies derived from CFC assays, phase contrast microscopy with Zif-VP64-Ep1 and Zif-VP64-Ep2 vectors. Lower row: fluorescent microscopy reveal colonies only Transfected with Zif-VP64-Ep2 vector. (**c**) Documentation of eGFP expression in colonies derived from CFC assays from transfected CD34^+^/eGFP^+^ cells with Zif-VP64-Ep2. RT-qPCR in mRNA extracts from CFC culture colonies (14 days) derived from FACs sorted CD34^+^/eGFP^+^ transfected with Zif-VP64-Ep2 cells for γ-globin mRNA expression (colonies from CD34^+^ Zif-VP64-Ep2).Values were normalized against colonies from non transfected CD34^+^cells (colonies from non transfected CD34^+^), plotted as 1.

Vector Zif-VP64-Ep1 transfected CD34^+^ cells with transfection efficiency of 24.2 ± 2.4% (Table 1), measured 48 hours post transfection, in total cells surviving transfection (Fig. 5a). Total RNA from these transfected cells was used for the estimation of relative γ-globin mRNA by RT-qPCR, 72 hours post transfection, and was found to have statistically significant increase, 2.7 ± 0.6% fold, compared to the γ-globin mRNA in non transfected CD34^+^ cells (Fig. 5b). CD34^+^/eGFP^+^ cells, sorted by fluorescence-activated cell sorting (FACS), were placed in colony-forming cell culture (CFC) in order to investigate their capacity for colony formation and the extent of plasmids’ retention among the colonies produced. Colonies appeared in these cultures, but they were all non-fluorescent (Fig. 6b and Table 1). This is rather expected, as, in CD34 + cells, promoter CMV does not support transcription of the eGFP gene and through the S/MAR element, which is a necessary condition for the S/MAR element to exert its plasmid retention function, as shown in previous studies^8^. Consequently, vector Zif-VP64-EP1 was not used as a control for the estimation of γ-globin induction in CD34 + cells, in CFC cultures.

The second episomal vector Zif-VP64-Ep2, carrying the ‘IR’ element along with the transcription cassette SFFV-eGFP-S/MAR, carrying the permissive for CD34 + cells promoter SFFV, was used to transfect similar CD34^+^ cells, with transfection efficiency of 23.3 ± 1.7% (Fig. 6a). FACS sorted CD34^+^/eGFP^+^ cells placed in Colony Forming Cell (CFC) assay, a semi-liquid culture with cytokines, giving rise to BFU-E and CFU-GM differentiated colonies from CD34 + hematopoietic progenitor cells (Table 1).

**C**FC cultures, 14 days post sorting, gave colonies that were all fluorescent (Fig. 6b and Table 1) in all four replicate cultures studied (Table 1). Estimation of the γ-globin mRNA in these colonies gave a statistically significant increase of 2.11 ± 0.13 fold (Fig. 6c) compared to that of the non transfected CD34^+^ cells.

These data show once more, that the S/MAR element must be present and furthermore it must also be transcribed in order to perform its plasmid retention function. And specifically they show that an episomal vector containing both elements, namely a transcribed S/MAR and the IR element, is capable of generating 100% transformed, fluorescent, colonies from colony forming cells (in total 17 days) derived from transfected haematopoietic, progenitor CD34^+^ cells.

## Discussion

Vectors based on S/MAR and IR chromosomal elements have been developed, able to transfect K562 cells, murine β-YAC cells and the human haematopoietic cells CD34^+^ with efficiencies that are satisfactory and within the range expected for their size^55,56^. These vectors (a) portray all properties of episomal vectors and (b) mediate efficient production of the synthetic, specific and functional γ-globin activator in all cells tested.

- Episomes, that are capable for mediating long-term nuclear retention of plasmid and transgene expression and exist in the cell as free, circular molecules without detectable integration into the cell’s chromosomes, are considered as competent episomes, in the sense that they have aquired the status of an autonomous replicon that replicates once per cell cycle with mitotic stability, segregating to daughter cells during successive mitoses^6,7^. The efficiency of plasmid retention varies and may depend on the level of transcription through the S/MAR element^57^. Evidence that the vectors described, are competent episomes derives from experiments of their transfer into K562 and CD34^+^ cells.
  Stable transfections in K562 cells with vector Zif-VP64-Ep1 showed that the S/MAR element is a necessary part of the competent vector, as stable transfections were not generated with control vector Zif-VP64-eGFP lacking the S/MAR element. The combined data from plasmid rescue assay and FISH analysis show that vector Zif-VP64-Ep1 has retained the episomal status of free, circular molecules in the transfected K562 cells, throughout the 28 weeks of culture, with no integration detected. Plasmid integration into the genome of eukaryotic cells is a rare event, considered to be initiated by double strand breaks, resulting in the formation of multimeric concatemers, that can be inserted into the genome by a ‘synthesis-dependent, microhomology-mediated end joining’ (SD-MMEJ) repair mechanism, by a process not well understood as yet^58,59^. Within this context, the lack of detection of integration of vector Zif-VP64-Ep1 implies that the vector’s DNA does not facilitate such a process. In the same stable transfections, it was calculated and there were 2.4 ± 0.2 plasmid copies per cell. In the FISH analysis of such cells, shown in Fig. 2d, only one copy of the plasmid is detected, while 2 copies of plasmid per cell are detected in Fig. S2 in the Supplementary information. This copy number value is within the range expected for the S/MAR based vectors, estimated between 2 and 12 copies per cell^56^, with the lower percentages referring to greater size vectors. The value estimated here, refers to stably transfected K562 cultures selected with G418. However, no drastic differences are expected on the copy number per cell between cultures with and without G418, mainly on the basis of eGFP expression during continuous, 28 weeks culture (Fig. 2b).
  These data lead to the conclusion that in human, heamatopoietic K562 cells, the level of fluorescence recorded and the level of transgene expression, as well as the calculated copy number of vectors per cell, represent the outcome of functioning, autonomously replicating, circular episomes. This is of special significance, as these two properties, combined, namely the non integration and the existence in free, circular plasmids constitute the cardinal requirements that must be fulfilled for an episome, in order to be a valid alternative to viral vectors.
  Transfections of CD34^+^ cells with vector Zif-VP64-Ep1 were transient, as FACS sorted CD34^+^/eGFP^+^ cells generated colonies in CFC assays, but none of them was fluorescent after 14 days of culture. Non fluorescent colonies do not carry vector copies, presumably because transcription through the cassette CMV-eGFP-S/MAR has been gradually diminished and as a result, S/MAR ceased to perform its plasmid retention function^8,9^.
  The second vector, Zif-VP64-Ep2, contains the transcription cassette SFFV-eGFP-S/MAR, with SFFV promoter ensuring transcription in CD34^+^. Sorted CD34^+^/eGFP^+^ cells, deriving from CD34^+^ transfections with vector Zif-VP64-Ep2, generated colonies in CFC assays, that were all fluorescent. This important result, namely 100% fluorescent colonies in CFC cultures, has also been documented in a previous study^9^ Specifically, in CD34^+^ transfections with a vector (pEPI-SFFV, without the IR element) that carries the same transcription cassette SFFV-eGFP-S/MAR, supported the generation of colonies in CFC assay, only 50% of which were fluorescent, verifying that the S/MAR not only should be present, but it should also be transcribed in order to support nuclear retention of the plasmid. In the same work, another vector (pEP-IR) containing the same transcription cassette SFFV-eGFP-S/MAR as well as the IR element, supported the generation of colonies in CFC cultures that were all (100%) fluorescent^9^. Exactly this result is also documented in the work reported here, with 100% of the fluorescent colonies that were generated from cells carrying vector Zif-VP64-Ep2. In both cases, it is the coexistence of a transcribed S/MAR element with the IR element in the same vector that accompanies complete retention of the vector and transgene expression in 100% of the colonies derived from the transfected CD34^+^.
  The appearance of colonies in CFC cultures *per se* does not, strictly speaking, signify long-term retention of the vector, which needs to be verified in long-term culture. Nevertheless, it denotes that the vector has reached the state of autonomous replicon, that facilitates vector establishment leading to stable transformation, by processes largely unknown and still under investigation.
  It is thus documented that the vectors Zif-VP64-Ep1 and Zif-VP64-Ep2 are competent episomes in conditions that ensure transcription through the S/MAR element. Furthermore, vector Zif-VP64-Ep2 carrying, in addition, the IR element is capable of producing colonies in CFC culture that are all fluorescent, which shows that it is fully competent for gene transfer into CD34^+^.
- Evidence that the vectors described, mediate efficient production of the synthetic, specific and functional γ-globin activator derives from their transfer all three types of cells tested.

The capacity of vectors Zif-VP64-Ep1 to support the production of the trans-acting factor VP64 was documented in stable transfections of K562 cells at the mRNA and the peptide level. The vector acts as specific γ-globin activator in K562 cells and the murine β-YAC cells (Figs. 3 and 4). γ-Globin mRNA production in β-YAC cells was detected in triplicate, quantitative real time PCRs and the determined relative amount against that of human β-globin and mouse α-globin ones in the same cells show that the estimated level can be of therapeutic significance. Furthermore, this experiment verifies results obtained with the same cells and the same activator acting on silenced, heterochromatin state, previously reported with viral transfer^47^.

Both vectors, Zif-VP64-Ep1 and Zif-VP64-Ep2, can activate γ-globin production in CD34^+^ cells, in transient state and in CFC colonies from CD34^+^/eGFP^+^ cells, respectively. This activation results in over 2-fold increase of γ-globin mRNA compared to the untransfected cells in all cases, and can be considered of therapeutic significance.

Two points of concern are the *specificity* of vector binding and the possible *toxicity* caused by the vector used.

Firstly, the gg1-VP64 trans-acting factor has been designed and developed by theoretical analysis to have a high *specificity* of binding to the target substrate, after viral transfer in CD34^+^ cells^46^. This was documented here for vector Zif-VP64-Ep1 in stably transfected K562 cells by ChIP assay, and it was found that the peptide VP64 is selectively binding onto target sequence gg1, in the ^A^γ promoter.

Secondly, a mild degree of *toxicity* is reported as a modest, initial reduction in cell accumulation in gg1-VP64 lenti-virally transduced cultures of erythroblasts from CD34^+^ cells, deriving from normal as well as from thalassaemic patients^50^. This was attributed to high expression of the transcription cassette for gg1-VP64, at the start of the transduced culture, resulting in excess of the peptide present. The use of the milder, erythroid specific, β-spectrin promoter provided lower level of *toxicity* without affecting induction of HbF. In our study, no reduction of growth was documented in K562 cells transfected with vector Zif-VP64-Ep1 containing the transcription cassette CMV-Zif-VP64.Additionally, the copy number per cell in these cultures, estimated at the level of 2.4 ± 0.2 per cell, is at the low side of the spectrum and it is not expected to contribute to very high expression of the transgene.

It is, therefore, shown that the episomal vectors Zif-VP64-EP1 and Zif-VP64-EP2 are capable for mediating endocellular production of a potent activator VP64, followed by the activation of the γ-globin gene in its native position, in all three types of cells tested. These results are reproducing faithfully the ones previously obtained with the use of viral vector^3,46,47,50^.

These data may derive from a synergy that may exist between the S/MAR and *β-globin Replicator* in the episomal vectors, as the chromosomal elements S/MAR and *β-globin Replicator* (IR), both deriving from the human genome, bring about specific properties to the episomal vectors.

The S/MAR, an element that has been studied extensively, is known to mediate binding of the episome to the nuclear matrix protein SAF-A, facilitating retention of the episome, which thereby, may acquire the status of an autonomous replicon, followed by vector establishment^60,61^. Recently, the contact sites of exogenous, episomal DNA in the genome of HeLa cells were mapped and characterized, using S/MAR-based replicons^62^. It was documented that the genomic contact sites are influenced by genomic *cis*-acting sequences that are incorporated in the replicon, and are in proximity to potential origins of replication.

The IR, on the other hand, a *bone fide* mammalian origin of replication, is a newly used chromosomal element, studied, so far, as part of three different episomal vectors all carrying an S/MAR element. Firstly, it was found that the transfer of vector pCEP4/CNV-eGFP-S/MAR into Jurkat cells failed to give stably transfected cell pools, but the insertion of the IR element into this vector, allowed the formation of stable transfections, through the exertion of strong chromatin opening activity facilitating DNA replication^45^. Secondly, the IR inserted into vector pEP-IR (SFFV-eGFP-S/MAR) used for transfection of haematopoietic CD34^+^ cells, raised the number of fluorescent colonies in CFC assay of FACS sorted CD34^+^/eGFP^+^ cells to 100%, from 50% that were in the absence of the IR^9^. Thirdly, in the present study, vector Zif-VP64-Ep2, that carries the same transcription cassette SFFV-eGFP-S/MAR as well as the IR element, again all colonies in the CFC assay are fluorescent (100%).

Evidently, the presence in the same episome of the IR, a strong origin of replication, along with the S/MAR, a strong retention element of the plasmid, has a drastic, positive effect in the function of the vector. In the light of the data presented by Hagedorn *et al*. recently, we consider that the IR may act as a genomic, *cis*-acting sequence incorporated in the replicon, which may influence positively the function of S/MAR element in the same replicon. It is as if the co-existence of the S/MAR and IR in the same replicon creates a synergy between the two elements, that augments the possibility for the replicon’s establishment onto the specific nuclear compartment, upon which the vector lands after transfection. The IR, therefore, is a chromosomal element that definitely promotes the function of S/MAR based episomal vectors after gene transfer into haematopoietic cells.

A number of possibilities for further investigations are opening up for vector Zif-VP64-Ep2. Firstly, it would be of interest to see whether vector Zif-VP64-Ep2 can transfect iPS cells from β-Thalassaemia and/or SCD patients, as iPS cells are amenable to culture and they are currently used in a number of pre-clinical studies.

Secondly, pre-clinical studies with transfer of this vector into mouse model of disease in order to determine their potential for clinical translation are vital. However, a delivery issue exists as the level of transfection efficiency for plasmid transfer into mouse progenitor cells is inefficient^9^. A valid option to address this problem is the cloning of the episomal plasmid into the genome of non-integrating viral vector, in which the S/MAR has already been shown to confer long-term nuclear retention of the vector^18^. Additionally, plasmid DNA transfer may be promoted in a setting of enhancing electrotransfection for improved nuclear entrance by facilitated transport through the nuclear pores or by cell cycle synchronization^63^. Finally, redesigning the structure of the current vectors so as to produce shorter but equally efficient vectors may be a first, necessary step.

In conclusion, episomal vectors have been obtained for the artificial γ-globin gene activator that are capable for mediating γ-globin gene activation in cells of haematopoietic origin with a gene expression program that allows for the production of γ-globin peptide, such as the K562 cells and the progenitor cells CD34^+^, as well as cells in which the γ-globin gene is inactive, such as the murine β-YAC cells. The γ-globin activation in each case is reaching therapeutic levels, as is the case with viral vectors. The vectors used portray the properties of the episomal vectors that are necessary for safe and efficient gene transfer, such as long-term nuclear retention as free circular plasmids, long-term expression and no-detection of integration. Importantly, transfections in CD34^+^ cells revealed exceptionally positive results, supporting 100% fluorescence of colonies in CFC cultures. It is considered that these properties are conferred by chromosomal elements S/MAR and *β-globin Replicator* co-existing in synergy within the respective vector. These properties as well as the consideration that episomal vectors are easy and cost-effective to produce, make them an excellent candidate for the development of the next generation vectors for gene therapy purposes.

## Methods

### Human cells

CD34^+^ cells from umbilical cord blood, derive from Hellenic Cord Blood Bank of the Biomedical Research Foundation Academy of Athens (BRFAA) The protocol was reviewed and approved by the BRFAA institutional review board (IRB) and the Institutional Ethics Committee.

### Plasmid construction

Plasmid Zif-VP64-Ep1 (10.5 kb) (Fig. 1c) was created by digestion of the pEPI-eGFP vector (6.7 kb) (Fig. 1b) with the *NciI* restriction enzyme, which digests the plasmid upstream the CMV promoter and downstream the S/MAR element, to free the pCMV-eGFP-S/MAR cassette, bearing sticky ends. The deriving pCMV-eGFP-S/MAR cassette is ligated to the lineralized plasmid pcDNA3-Zif-VP64 (6.1 kb) (Fig. 1a), using restriction enzyme *NciI*, which digests the plasmid upstream the pSV40 ori.

Plasmid Zif-VP64-Ep2 (11,1 kb) (Fig. 1d) was created as follows. Firstly, a new, intermediate plasmid was made, deriving from the Zif-VP64-Ep1 vector by replacing the CMV promoter with the SFFV promoter (SFFV-Zif-VP64-Ep1). Plasmid SFFV- Zif-VP64-Ep1 was linearized by *DraIII* and the resulting molecule was made blunt-ended by treatment with Klenow enzyme and it was dephosphorylated by DNA phosphatase (Antarctic phosphatase, NEB). Then, the fragment with the ‘IR-SFFV-eGFP-S/MAR-SV40 polyA’ transcription cassette was removed from the pEP-IR vector^9^ by the action of the restriction enzymes *Mlu*I and *Pci*I, and was made blunt-ended by treatment with Klenow enzyme. Finally, the two fragments, SFFV-Zif-VP64-Ep1 and IR-SFFV-eGFP-S/MAR-SV40 polyA, were ligated and used for bacterial transformation (DH5-alpha *E.coli*). The resulting colonies were isolated and analyzed to obtain vector Zif-VP64-Ep2.

Control plasmid Zif-VP64-eGFP (8.1 kb) (Fig. 1e) is derived from the Zif-VP64-Ep1 (10.5 kb) by deleting the S/MAR element with *AccI* restriction enzyme that has recognition sites upstream and downstream the S/MAR element.

### Cell isolation, cell culture and transfection

Cells of the Human erythroleukemia K562 established cell line, were cultured in DMEM (Gibco) supplemented with 10% FCS, 2 mM L-glutamine, 50 U/ml penicillin and 50 mg/ml streptomycin. K562 cells were transfected by electroporation with the use of GenePulserII (BioRad Hemel Hempstead, UK). At least 1 × 10^6^ K562 cells were mixed with 10–15 μg of plasmid DNA, added to a 0.4 cm electroporation cuvette (BioRad, Hemel Hempstead, UK) and electroporated at 220 V, 975 mF (t: 24.1–26.7). After electroporation, cells were immediately added to 10 ml preheated complete medium and incubated at 37 °C in 5% CO_2_.Transfected cells were selected 24 hours after transfection by addition of Geneticin (G418) (Invitrogen, Carlsbad, CA) in the medium, at a concentration of 800 mg/ml. After 14–19 days, stably transfected and selected cells were split in two and were further cultured either with G418 (at a concentration of 400 mg/ml) or without G418.

β-YAC cells^47^ were cultured in IMDM (Iscove’s Modified Dulbeccon’s Medium) supplemented with 10% heat inactivated FCS, 2 mM L-glutamine, 1x HEPES, 1x MEM (Non Essential Amino Acids) and 3.16 mΜ demerization reagent AP20187 (ARIAD) as described before^46^. All reagents were purchased from Invitrogen (Carlsbad, CA). β-YAC cells were transfected with electroporation, with a GenePulserII (BioRad Hemel Hempstead, UK). Cell suspension 3.75 × 10^6^ up to a final volume of 500 μl was mixed with 10–15 μg of plasmid DNA, added to a 0.4 cm electroporation cuvette (BioRad, Hemel Hempstead, UK) and electroporated at 250 V, 350 μF (t: 07.8–09.8 msec). After electroporation, cells were immediately added to 5 ml preheated complete IMDM, medium and incubated at 37 °C in 5% CO_2._ β-YAC cells ware also transfected with the help of the ¨*in vivo* Transfection Reagent ExGen500¨ (Fermentas) using 2 × 10^6^ β-YAC cells and 10–15 µg 10–15 μg of plasmid DNA in NaCl 150 mM at a final concentration of 10 μg/ml as described before^46^. Both techniques for β-YAC cells transfection were successful.

CD34^+^ cells were isolated from low-density mononuclear cells (MNC), by gradient isolation (1.077 g/ml Ficoll-Paque, Biochrom AG, Germany) and immunomagnetical selection, using a combination of the Miltenyi CD34 MultiSort kit (Miltenyi Biotec, Gladbach, Germany) and the LS Columns (Miltenyi Biotec, Bergish Gladbach, Germany) in accordance with the manufacturer’s instructions. In all preparations, CD34^+^ cell frequency exceeded 98% as counted by flow cytometry. CD34^+^ cells were cultured at a concentration of 2–5 × 10^5^ cells/ml in IMDM, with 10% FCS supplemented with the recombinant human (rh) cytokines: 20 ng/ml rh stem cell factor (SCF), 50 ng/ml rh thrombopoietin (TPO) and 50 ng/ml rh Flt-3 ligand (FL) (complete serum-free medium). Cell culture reagents were purchased from Invitrogen (Carlsbad, CA) and recombinant cytokines were purchased from BioSource (Nivelles, Belgium). CD34^+^ cells were transfected by nucleofection, using the Amaxa NucleofectorII devise (programme U008) with Human CD34^+^ Cells Nucleofector solution (Amaxa, Koeln, Germany) following the manufacturer’s instructions.

### Fluorescence microscopy, flow cytometric analysis and cell sorting

Expression of the reporter gene eGFP in the tranfected cells was observed by fluorescence microscope (Nikon eclipse TE 2000), using the fluorescein isothiocyanate dichroic filter set as filter cube, (excitation at 450–490 nm; emission at 520 nm).

Approximately 3–5 × 10^5^ cells, after wash twice with 1x PBS, were analyzed on an EPICS-XL flow cytometer (Coulter, Miami, FL, USA). Approximately 5 × 10^5^ cells were washed and incubated with an anti-CD34-phycoerythrin (PE)-conjugated monoclonal antibody (Pharmingen, BD, Erembodegen, Belgium) for 30 min at 4 °C. Nonviable cells were excluded by gating based on propidium iodide (PI) (Pharmingen, BD). Transfected cells were sorted with a FACS Vantage (BD, Erembodegen, Belgium) as previously described^8,64^. Times and conditions for the estimation of eGFP and γ globin expression for each cell type is provided in the respective Results Section.

### Fluorescence *in situ* hybridisation (FISH)

K562 cells stably transfected with the Zif-VP64-Ep1 (10.5 kb), were hybridized with pcDNA3-Zif-VP64 (6.5 kb) plasmid, used as a hybridization probe for the detection of episomal and/or integrated transgenes, labelled with Fluorescein-12-2′-deoxy-uridine-5′-triphosphate (Roche). The procedure is reported in detail previously^9^. Probe labelling was performed by Nick Translation Mix (Roche) following the manufacturer’s instructions. For internal control, DLEU (13q14) hybridization probe was used, labelled with Fluorescein-12-2′-deoxy-uridine-5′-triphosphate (Roche) as well as Tetramethyl-Rhodamine-5-dUTP (Roche) that detects the endogenous 13q14 locus giving a double -green and red–signal respectively as reported previously^64^. A total of 50 metaphase and interphase spreads were analysed.

### Plasmid rescue assay

Extrachromosomal DNA was isolated from 90-120 colonies from CFC assay, randomly collected, pooled and washed in 1x PBS as previously described with a modified HIRT protocol^8^. The HIRT extract was used for transformation of *E. coli* ElectroMax DH5a-ETM cells (Invitrogen, Carlsbad, CA) by electroporation, according to manufacture’s instructions and the transformed *E. coli* were selected using LB-agar plates containing 30 μg/ml kanamycin (Invitrogen, Carlsbad, CA). Plasmid DNA that was extracted (QIAprep Spin Miniprep Kit, Qiagen, Hilden, Germany) from single resistant colonies, randomly selected was subjected to restriction enzyme analysis by *NotI*, *NotI*x*PciI*, and *NheI* restriction enzymes.

### DNA extraction and Plasmid Copy Number estimation in K562 cells

Total DNA from 1 × 10^6^ K562 cells, transfected with Zif-VP64-Ep1 and cultured in the presence of G418, was isolated with AllPrep DNA/RNA/Protein Mini Kit (Qiagen) and subsequently treated with RNase I (Promega). 100 ng of total DNA was amplified using QuantyiFast SYBER Green PCR (Qiagen) and the LightCycler2.0 (Roche) as follows: 10 sec at 95 °C, 30 sec at 60 °C, 40 cycles. The eGFP DNA primers used were: eGFP_F 5′-GAC CAC TAC CAG CAG AAC AC-3′ and eGFP_R 5′-GAA CTC CAG CAG GAC CAT G-3′ (Invitrogen). Copy number estimation procedure was carried out using the Absolute Quantification analysis (Light Cycler Software 4.05, Roche) as reported previously in detail^9^.

### RNA extraction, RT-PCR and Real time –quantitative PCR

RNA was extracted from 1 × 10^6^ K562 cellswith RNeasy^®^ Mini Kit (Qiagen). The extracted RNA was incubated for 1 h at 25 °C with DNaseI (RNase free) (TAKARA) and cleaned up with RNeasy^®^MinEluteCleanup Kit (Qiagen) in order to clear the remaining DNA. 800–1000 ng RNA was reverse transcribed to cDNA with PrimeScriptTM, 1^st^ strand cDNA Synthesis Kit with Oligo dT primers (Takara).

Primers, used in the PCR reaction for the detection of the Zif-VP64 cDNA, were: TF-1: 5′-ACTGCCGCGACCTTGCT-3′ and TF-2: 5′-CGGAACGTCGTACGGGTAGTT-3′ (Τm = 55 °C), and nested primers TF-3: 5′-TTCTCCCGCAGCGATCAC-3′and TF-4: 5′-CCAAAGCACCTGGGTCTGA-3′ (Τm = 55 °C). Primers for the human endogenus GAPDH gene GAPDH_F: 5′-CCATGTTCGTCATGGGTGTGA-3′and GAPDH_R: 5′-CATGGACTGTGGTCATGAGT-3′ (Τm = 57 °C) as described before^46^.

The PCR reactions were performed using 0.25 u Taq polymerase (Invitrogen, Carlsbad, CA), 0.5 pmol dNTPs (Fermentas, Hanover, MD) and 0.25 pmol of each specific primer (Invitrogen, Carlsbad, CA).

For the detection of γ-globin ggRNA, One Step RT-PCR Kit (Qiagen) was used, with primersggRT_F:5′-GACAAGCTGCATGTGGATCCT-3′, and ggRT_R: 5′-CCGAAATGGATTGCCAAAAC-3′ (Τm = 56 °C) as described before^46^. For Real-time PCR, dilutions 1/10 and 1/100 cDNA were amplified using QuantyiFast SYBER Green PCR (Qiagen) and the LightCycler2.0 (Roche) as follows:10 sec at 95 °C, 30 sec at 60 °C, 40 cycles.

(Moa-globin): 5′-GATTCTGACAGACTCAGGAAGAAAC-3′and 5′-CCTTTCCAGGGCTTCAGCTCCATAT-3′, the human β-globin (Hub-globin): 5′-ACACAACTGTGTTCACTAGCAACCTCA-3′and 5′-GGTTGCCCATAACAGCATCAGGAGT-3′, the human γ-globin (Hug-globin): 5′-GACAAGCTGCATGTGGATCCT-3′and 5′-CCGAAATGGATTGCCAAAAC-3′and the mouse GAPDH gene (ΜοGAPDH): 5′-AGGCCGGTGCTGAGTATGTC-3′και 5′-TGCCTGCTTCACCACCTTCT-3′.

### Chromatin immunoprecipitation-ChIP

For the chromatin immunoprecipitation (ChIP) procedure, the EZChIP ™ Chromatin Immunoprecipitation Kit (Upstate) was used according to the manufacturer’s instructions. The process includes the following steps: i. fixation of all transcription factors bound to the DNA prior to cell lysis, by treatment with 37% formaldehyde (SIGMA); ii. lysis of approximately 1 × 10^7^ transfected and equal amount of non-transfected K562 cells and isolation of purified chromatin; iii. Sonicatoion, in Sonics & Materials Inc. -Vibra Cell Ultrasonic Probe Power Generator POWER at 120 vac, 25 hz, 2 amp on ~500 bp portions on average; iv. immunoprecipitation of the protein/DNA complex using 10 μl of the specific anti-ZIF-VP64 antibody. As a control, an anti-histidine antibody, provided by the Chip kit, was used. DNA was cleared from the antibody/protein/DNA complex, purified and used as a template in PCR procedure, with specific primers for the human γ-globin promoter: γ-ChIP_F: 5′-GACTGAATCGGAACAAGGCAAAG-3′, γ-ChIP_R: 5′-TGTCCTCCTCTGTGAAATGACCC-3′ (Tm = 60 °C) and for the human GAPDH control gene: G-ChipF: 5′-TACTAGCGGTTTTACGGGCG-3′, G- ChipR: 5′-TCGAACAGGAGGAGCAGAGAGCGA-3′ (Τm = 57 °C).

### Western blotting

Total cell lysates were prepared by lysing cell pellets directly in SDS/PAGE loading buffer (50 mM Tris-HCl pH 6.8, 2% SDS, 10% glycerol, 50 mM dithiothreitol and 0.1% bromophenol blue) and boiling. All protein extracts were resolved in a 10% SDS-PAGE gel and electrotransferred to a polyvinylidene difluoride (PVDF) membrane (Millipore, Bedford, MA). γ-Globin protein was detected with anti-γ-globin rabbit antibody 1/500 (SantaCruz Biotechnology, SantaCruz, CA) and the endogenous tubulin was detected with anti-tubulin mouse antibody 1/10.000 (Sigma-Aldrich Chemical Co., St. Louis, MO).

### Intracellular staining

For flow cytometric detection of HbF (66.7 kDa) (intracellular staining, IS) phycoerythrin-conjugated monoclonal anti-HbF-PE (Phycoerythrin, PE) antibody (Caltag Laboratories, Burlingame, CA) was used according to instructions for intracellular staining. Transfected K562 5 × 10^5^ cells, after culture with and without selection pressure, as well as non transfected cells were suspended in 1x PBS, incubated with Cytofix/Cytoperm chemist (PharMingen) for 20 min at 40 °C, and then vigorously vortexed in duplicate with BD 1 xPerm/Wash Buffer (BD Biosciences). Consequently, monoclonal anti-HbF antibody was added and cells were incubated for 30 min at 40 °C, followed by a wash with BD 1x Perm/Wash Buffer and analysis of the cell suspension and quantification of HbF was by flow cytometry (using FACS)^65,66^.

### Statistical analysis

Statistical analysis was performed using Student’s t test and p values less than 0.05 were considered statistically significant.

## Supplementary information

Supplementary Information
