# Supplementary material for: Episomal vectors based on S/MAR and the β-globin Replicator, encoding a synthetic transcriptional activator, mediate efficient γ-globin activation in haematopoietic cells
Source: Sci Rep. 2019 Dec 24;9:19765. doi: 10.1038/s41598-019-56056-z (PMC6930265; doi:10.1038/s41598-019-56056-z)
Supplement: Supplementary file 1 — Supplementary Information [file 41598_2019_56056_MOESM1_ESM.pdf]

Supplementary Information

Title:

Episomal vectors based on S/MAR and the β-globin Replicator, encoding a synthetic transcriptional activator, mediate efficient γ-globin activation in haematopoietic cells

Authors list:

Eleana F. Stavrou, Emannuel Simantirakis, Meletios Verras, Carlos Barbas III, George Vassilopoulos, Kenneth R. Peterson, and Aglaia Athanassiadou

Suppl Figure S1. K562 cells transfected with vector Zif-VP64-eGFP

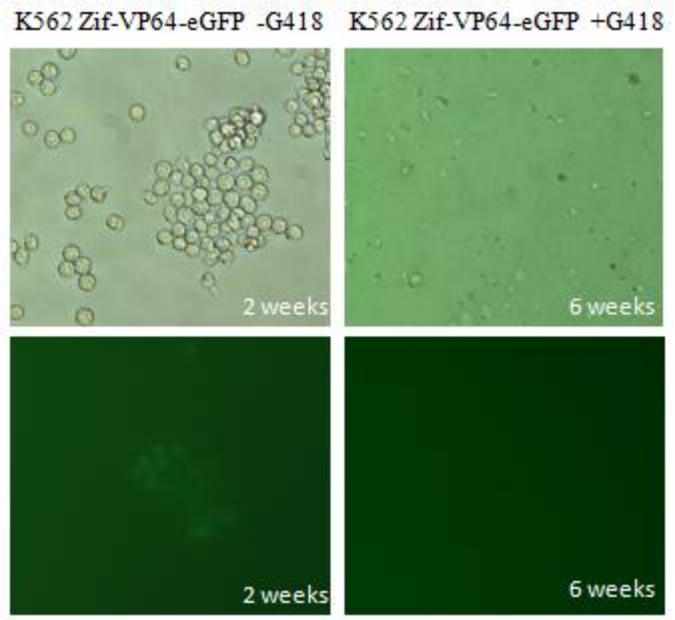

Suppl. Figure S1: K562 cells transfected with Zif-VP64-eGFP (left panel photos), were selected with 800 micrograms/ml G418 for 2 weeks after transfection, and at this point selection was withdrawn and a -G418 culture started (right panel photos). At 6 weeks post transfection, 400 micrograms/ml G418 were added and cells died out. Results are shown from phase contrast (upper column) and fluorescent microscopy (down column).

Suppl.Figure S2. FISH analysis of K562 cells transfected with vector Zif-VP64-Ep1

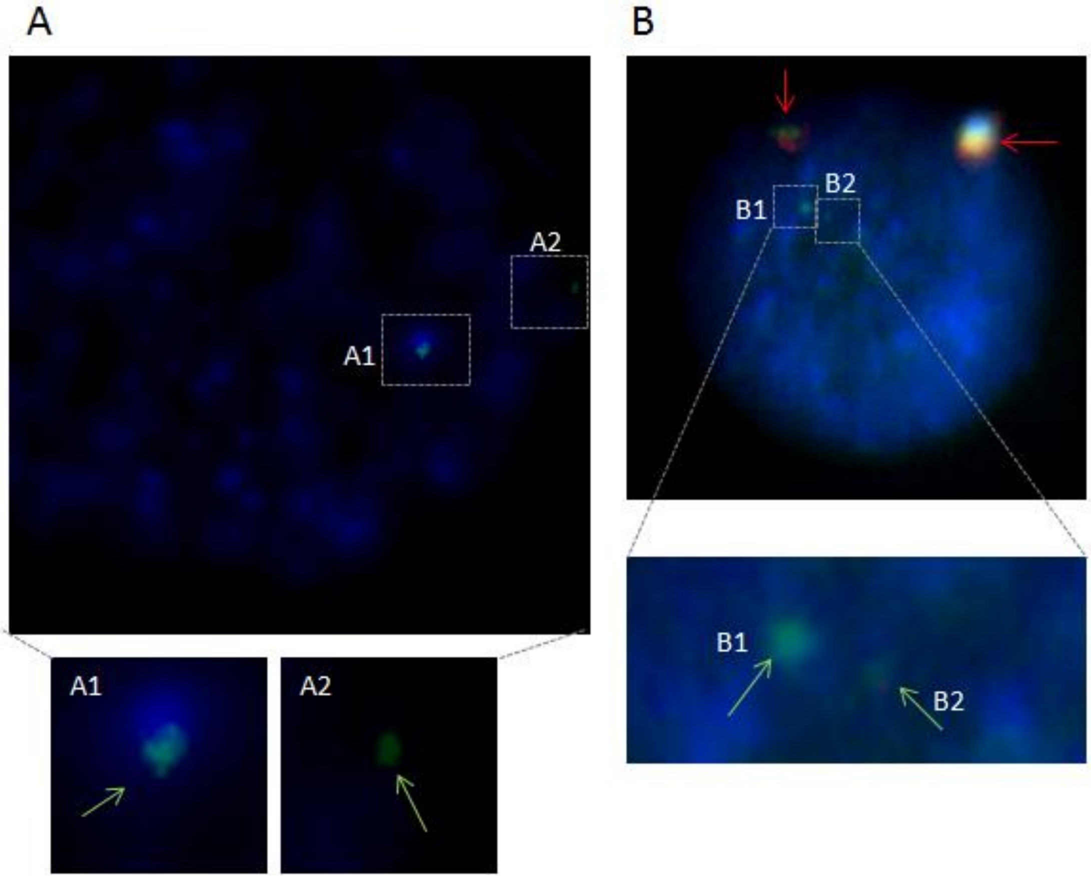

Suppl. Figure S2: Cells at 20 weeks of continuous culture supplemented by 400mg/ml G418. Green arrows show Zif-VP64-Ep1 plasmid as episomes. Red arrows show the endogenous 13q14 locus, used as control, giving a double -green and red–signal. Information on labelling is in Materials and Methods, paragraph on FISH.

(A) Representative interphase. A1 and A2 show Zif-VP64-Ep1 plasmid episomes (2 copies); (B) Representative metaphase spreads, B1 and B2 show Zif-VP64-Ep1 plasmid episome (2 copies).

Suppl Figure S3. γ-globin and tubulin detection in Western blot analysis (Fig. 3d)

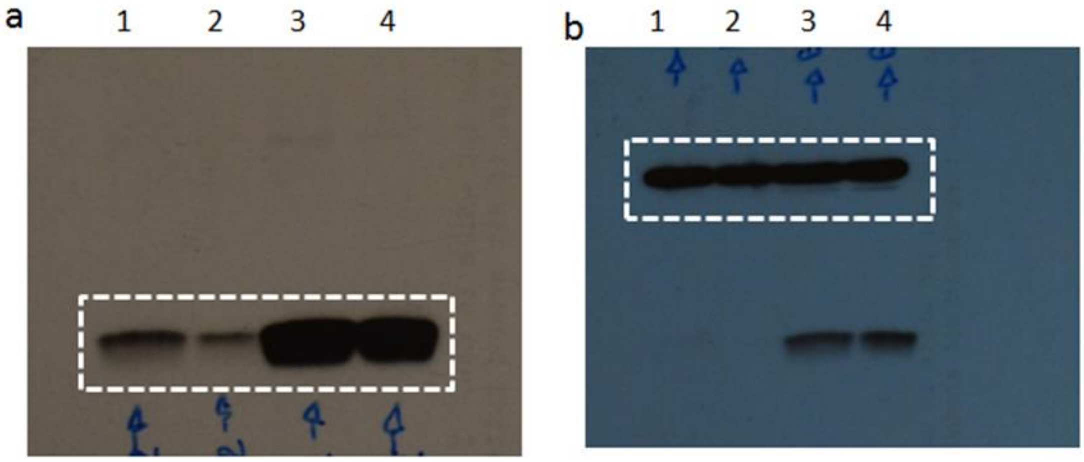

Suppl. Figure S3: (a) γ-globin and (b) tubulin detection in K562 untransfected cells (1, 2) and in K562 cells transfected with Zif-VP64-Ep1 (3) 5 weeks and (4) 15 weeks period of continuous culture.

Suppl Figure S4. Expression γ-globin, a-globin and GAPDH in β-YAC non transfected cells

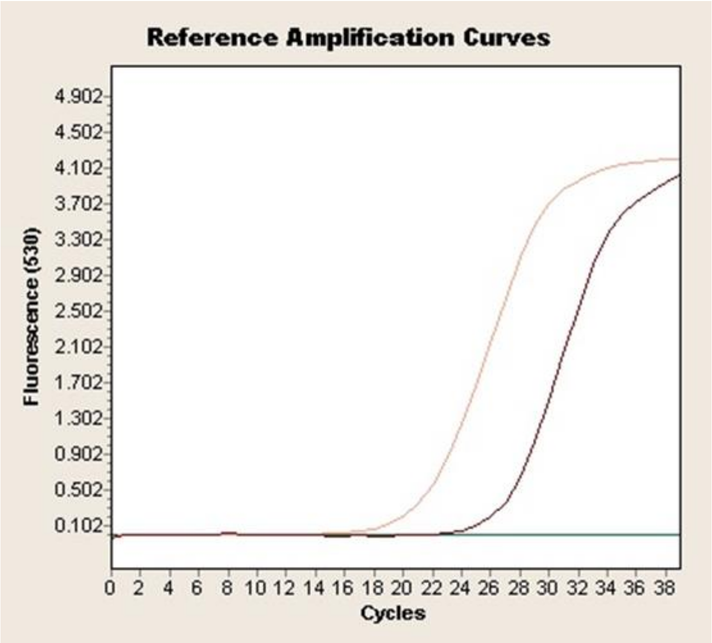

Suppl.Figure S4: (orange) α-globin, (red) GAPDH and (green) γ-globin (flat line as non expressed) expression profile in β-YAC non transfected cells .
